# Multifunctional, Biocompatible Hybrid Surface Coatings Combining Antibacterial, Hydrophobic and Fluorescent Applications

**DOI:** 10.3390/polym17152139

**Published:** 2025-08-05

**Authors:** Gökçe Asan, Osman Arslan

**Affiliations:** Food Engineering Department, Istanbul Sabahattin Zaim University, 34303 Istanbul, Türkiye; asangke33@gmail.com

**Keywords:** multifunctional coating, antibacterial, hydrophobic, hybrid, quantum dot

## Abstract

The hybrid inorganic–organic material concept plays a bold role in multifunctional materials, combining different features on one platform. Once varying properties coexist without cancelling each other on one matrix, a new type of supermaterial can be formed. This concept showed that silver nanoparticles can be embedded together with inorganic and organic surface coatings and silicon quantum dots for symbiotic antibacterial character and UV-excited visible light fluorescent features. Additionally, fluorosilane material can be coupled with this prepolymeric structure to add the hydrophobic feature, showing water contact angles around 120°, providing self-cleaning features. Optical properties of the components and the final material were investigated by UV-Vis spectroscopy and PL analysis. Atomic investigations and structural variations were detected by XPS, SEM, and EDX atomic mapping methods, correcting the atomic entities inside the coating. FT-IR tracked surface features, and statistical analysis of the quantum dots and nanoparticles was conducted. Multifunctional final materials showed antibacterial properties against *E. coli* and *S. aureus*, exhibiting self-cleaning features with high surface contact angles and visible light fluorescence due to the silicon quantum dot incorporation into the sol-gel-produced nanocomposite hybrid structure.

## 1. Introduction

Multifunctional materials are functional structures that combine multiple and improved properties for different applications, providing innovative nanocomposites by tailoring the different requirements [1]. Among them, hybrid surface coatings that exhibit polymeric features and glass-ceramic-like properties can be fused within a new concept addressing the advantages of organic polymers and ceramic properties [2]. For example, organically modified ceramics can be applied to glass, metals, wood, and bio-based plastics and fibres like paper and textiles [3]. Since multifunctional materials are advanced engineered substances formed to perform multiple functions simultaneously, often in response to environmental stimuli, it is vital to combine diverse properties such as mechanical strength, antibacterial features, thermal regulation, electrical conductivity, and responsiveness to light, pH, or stress [4]. The key point here is to integrate the necessary functionalities at the molecular or structural level, enabling the development of systems that are lighter, productive, more efficient, multiresponsive, and adaptive [5]. Therefore, a systematic approach from innovations in material synthesis, such as bottom-up or top-down, composite engineering, bio-inspired design, sol-gel technique, and gas phase synthesis, must be selected to tailor properties to meet specific applications [6]. It was widely experienced that advanced materials such as nanoporous composites enable the material separation processes, which are vital for water purification, air filtration, and industrial waste treatment [7].

These materials, engineered with tunable pore sizes and surface chemistries, can efficiently remove contaminants while maintaining high flow rates and energy efficiency. Similarly, photocatalytic coatings on filters degrade organic pollutants under light exposure, offering self-cleaning capabilities and extended operational lifespans, which reduce maintenance costs and environmental impact [8]. In sensing applications, multifunctional materials integrate responsiveness with high sensitivity to detect and respond to environmental changes [9]. Also, materials with tunable optical or electrical properties, such as quantum dots, metal-organic frameworks, or conducting polymers, are employed in chemical and biological sensors to detect specific analytes with high precision and reliability [10]. Generally, these sensors are crucial in healthcare for rapid diagnostics and industrial process control to optimise production systems, ensuring efficiency and safety in critical operations [11]. Multifunctional materials are pivotal in advancing medical devices, drug delivery systems, and biocompatible coatings. For example, hydrogels, designed with dual biocompatibility properties and responsiveness to stimuli such as pH and temperature, are widely utilised for controlled drug release systems, ensuring safe delivery systems and reducing side effects [12]. Antibacterial coatings, infused with silver nanoparticles, may prevent biofilm formation on medical equipment, implants, and surfaces in healthcare platforms, significantly lowering infection risks [13]. Multifunctional biomaterials may enhance catalytic efficiency and stability by immobilising different enzymes or biostructures on porous supports, accelerating biochemical reactions while ensuring robustness under harsh conditions. These platforms, spanning diverse sectors and applications, underscore the versatility and transformative potential of step-by-step produced multifunctional materials [14].

**Figure 1:**
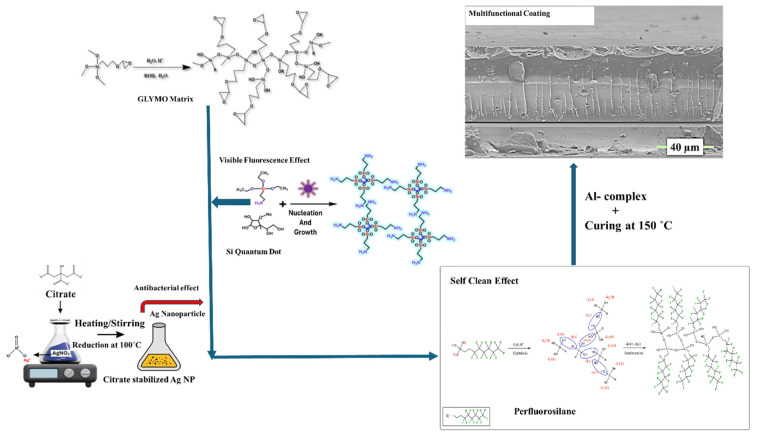
General representation of the multifunctional biocompatible coating combining antibacterial, fluorescent, and hydrophobic concepts.

Putting the most valuable efforts together and combining the different material features on one matrix, the concept of multifunctional materials represents a paradigm shift in materials chemistry, wherein the integration of diverse properties into a single system is achieved through precise molecular design, advanced fabrication techniques, and a deep understanding of structure–property relationships [15]. These materials exemplify chemistry, physics, and engineering convergence, enabling solutions that address complex challenges. Also, application-driven interplay of nanoscale features, hierarchical architectures, and tailored chemistry allows multifunctional materials to simultaneously exhibit properties such as mechanical robustness, thermal stability, electrical conductivity, and environmental responsiveness [6]. This synergy enhances their efficiency and performance and fosters resource and energy savings, aligning with sustainability goals in the near future. Using this approach, we have developed a step-by-step production of biocompatible surface coating material (Figure 1) that combines the fluorescent visible light emission, antibacterial resistance against some harmful bacteria, and hydrophobic efficiency, providing the self-cleaning property on metal surfaces.

## 2. Materials and Methods

### 2.1. Materials

AgNO_3_ (≥99.0%), 3-Aminopropyl)triethoxysilane (99%), (3-Glycidyloxypropyl)trimethoxysilane (≥98%), and L-ascorbic acid (99%) were purchased from Sigma Aldrich (Massachusetts, USA). Tridecafluoroctyl triethoxysilan (FAS Dynasilan^®^ F8261) was purchased from Evonik (Essen, Germany), and trisodium citrate (99%) was purchased from Alfa Aesar (Haverhill, MA, USA), and they were used without further purification. Technical ethanol and acetone were used in cleaning/drying procedures.

### 2.2. Characterisation

X-ray photoelectron spectroscopy (XPS) spectra were obtained by a flood gun charge neutraliser system equipped with a monochromated Al Ka X-ray source (hʋ = 1486.6 eV) from a 400 mm spot size on the nanofibrous web. Wide energy survey scans were recorded in the 0–1360 eV binding energy range, at a detector pass energy of 200 eV, and with an energy step size of 1 eV. High-resolution spectra were obtained at a pass energy of 50 eV with energy steps of 0.1 eV for each atom. The elemental composition investigations (EDX mapping) of the obtained nanoparticles and nanocomposite layers were performed together with morphology and thickness analyses with a scanning electron microscope (SEM) (Quanta 200 FEG, FEI). In order to avoid the electron charging effect, the surface of the coatings was coated with 5 nm Au prior to the SEM imaging. Sometimes, this procedure was not applied to the second measurements. X-ray diffractometer (XRD) (STOE-STADI MP) was used to determine the X-ray diffraction pattern of the nanocomposite coating in the powder form with Cu Ka radiation in the 2θ = 10–80 range. Additional morphological, surface, and thickness examinations were conducted using an EDS by transmission electron microscopy (TEM, Tecnai G2 F30). The same equipment detected HR-TEM images of the silicon quantum dots. The optical behaviour of the obtained nanoparticles and quantum dots was observed by UV-vis spectrophotometry (Optima SP 3000) using a fluorescence spectrophotometer (Horiba, Fluoromax 4). The materials’ thermal features and synthesised coatings were compared between room temperature and 900 °C with Seiko EXSTAR SII 6300 TGA-DTA. A nitrogen atmosphere was used as the decomposition environment.

Biocompatibility of surface coating on the L929 cell line viabilities was determined by the MTT colorimetric assay. Each cell line was seeded at a density of 1 × 10^4^ cells per well in 96-well plates and treated with nanocomposite coating exposure for 24, 48, and 72 h. Then, 10 μL of MTT dye (5 mg/mL in PBS) was applied to each well for four hours. Due to mitochondrial enzyme activation, formazan crystals converted from MTT dye were solubilised by 100 µL of dimethyl sulfoxide (DMSO). The absorbance of the suspension was measured at 590 nm by a Biotek 800 TS Microplate reader. The antibacterial activity against *S. aureus* and *E. coli* was studied for the nanocomposite surface, which was coated on the glass surface, mimicking the disc diffusion technique. Inhibition zones were observed accordingly. Both bacteria were incubated in a growth medium with the nanocomposite coatings, and after the defined time ranges, inhibition rates were calculated using the inhibition diameters.

### 2.3. Synthesis Methods

#### 2.3.1. Synthesis of Silicon Quantum Dots (Si QDs)

To synthesise the surface-protected silicon quantum dots (Si QD), aminopropyl triethoxysilane (APTES) was dissolved in water, and a reducing agent, sodium ascorbate (99%), was introduced at a 1:4 proportion. The mixture was stirred for 3 min at 500 rpm and placed under UV light (Osram, Ultravitalux, 300 W, radiated power: 315–400 nm (UVA) is 13.6 W, and 280–315 nm (UVB) is 3.0 W, 15 cm length) under constant stirring. After 45 min of UV treatment, aliquots were taken for examination. The UV light source lamp was shut down for 15 min at 30-min intervals for cooling after 15 min. The solution transformed into a pale, red-wine colour, and its fluorescence feature was detected immediately.

#### 2.3.2. Prehydrolysis of the Fluorosilane Surface Agent

Tridecafluoroctyl triethoxysilan (Dynasilan^®^-F 8261-FAS) was prehydrolised with 0.1 M HCl per mole hydrolisable –OR group and stirred at room temperature for 24 h. After prehydrolysis, a straightforward solution for F silane was obtained. The initial ratio between the OR/H_2_O was 3/1 at the beginning of the hydrolysis, like GLYMO prehydrolysis. Then, the hydrolysis ratio was converted to 1/1 using 0.1 M of HCl. Obtained prehydrolised tridecafluoroctyl triethoxysilan (FAS) was then utilised to modify the final multifunctional coating.

#### 2.3.3. Synthesis of Ag Nanoparticles by the Turkevich Method

A typical silver nanoparticle (Ag NP) synthesis prepared 60 mL of 0.25 mM silver nitrate solution in a flask. Independently, 34.0 mM (1.0 wt%) trisodium citrate solution was prepared separately. The flask containing the AgNO_3_ solution was heated using a hotplate with constant and vigorous stirring (500 rpm). A cap was used to cover the flask for proper synthesis. A specific trisodium citrate solution was rapidly added after the AgNO_3_ solution reached the boiling point under ambient pressure. The molar ratio was always kept similar to obtain monodispersed nanoparticles since the Ag/trisodium citrate ratio is the key factor in achieving the desired particle size. Synthesis of Ag nanoparticles was complete when the colour of the suspension no longer changed. The solution was cooled and kept at 4 °C to prevent further adverse effects.

#### 2.3.4. Synthesis of the Blank Hybrid Coating by the Sol-Gel Technique Using the Spray Method

To an aluminium trisec-butoxide solution, an equimolar amount of ethylacetoacetate (EAA) was added, dropwise. The solution was stirred vigorously and cooled in an ice bath to prevent overwarming. Because of the exothermic nature of the complexation reaction, the solution should be kept around room temperature. After the complexation reaction, the solution was cooled to room temperature, and stirring was carried on. The complex aluminium structure allows us to co-condense with epoxysilane, leading to a hybrid compound. Therefore, 3-Glycidyloxypropyl trimethoxy silane (GLYMO) was prehydrolised with 0.1 M HCl per mole hydrolisable –OR group and stirred at room temperature for 6–12 h. After prehydrolysis, a clear solution was obtained. During hydrolysis, there was no observable difference. The initial ratio between the OR/H_2_O was 3/1 at the beginning of the hydrolysis. Then this ratio was converted to 1/1 using 0.1 M HCl while additional additive dispersion was carried out. After adding prehydrolised epoxysilane, 15 min of mixing was carried out, and the obtained structure was sprayed on aluminium surfaces with 4–6 bar pressure with an adjustable nozzle. Coatings were cured at 120 °C for 20 min. 

#### 2.3.5. Synthesis of the Multifunctional Hybrid Surface Coating via the Step-by-Step Method

For the multifunctional smart coating synthesis by a step-by-step method, 3% Si QD solution was added into the total weight of the MNK-1 coating, leading to the MNK-2 structure. Also, 12% of the prehydrolysed FAS solution was mixed with Si QD for the MNK-3 coating preparation. After these two components were added, as stated before, aluminium trisec-butoxide/ethylacetoacetate complex was added dropwise, and the solution was stirred vigorously and cooled in an ice bath. Smart coating can be applied by adding an aluminium complex into the 3-Glycidyloxypropyl trimethoxy silane (GLYMO), which was prehydrolysed with 0.1 M HCl per mole of hydrolisable –OR group. For the MNK-4 multifunctional coating, silver nanoparticles (1% according to the total weight), which were obtained by the Turkevich method, were also introduced into the same solution (containing 3% Si QD and 12% FAS), and stirring and dispersion were carried on for about 30 min (500 rpm). After the molecular dispersion of these functional materials was completed, spray coating was applied on aluminium metal surfaces with 4–6 bar pressure with an adjustable nozzle. Coatings were cured at 120 °C for 20 min.

#### 2.3.6. Physical and Mechanical Properties of Multifunctional Hybrid Surface Coatings

The scratch and adhesion tests of the surface coatings were performed according to the cross-cut/tape test of the ASTM D 3359 [16]. In this test, coated surfaces are scratched with the tool of the test to form small squares on the surface (cross-hatched test). Then, the tape is adhered to the surface where little squares appear and are firmly pulled back. Scratch resistance of the coatings was determined with a multi cross cutter (Erichsen, type 295). Adhesion and scratch behaviour of the coatings were determined with a lattice cut/tape test (Erichsen, ASTM D 3359). Tests indicated that all coatings (MNK-1, MNK-2, MNK-3, MNK-4) have the 5B value as the test result for the scratch resistance. The adhesion of the coatings on metal substrates was also determined. All coatings’ GT/TT ratio was excellent, which means it was equal to zero. According to the test, it is already known that if adhesion is excellent, GT/TT = 0; if there is no adhesion between substrate and coating material, GT/TT = 5. This difference shows that all coatings have excellent adhesion on the surfaces. Adhesion and scratch tests proved that these multifunctional coatings adhere to metal surfaces without using any other material, enhancing the coatings’ adhesion level. Without using a primer (chemical compounds that increase surface functionality and adhesion), these coatings can be applied onto the metal surfaces to prevent abrasion, scratches, and possibly corrosion. The static water contact angles on the multifunctional coatings were evaluated using a contact angle analysing instrument (Theta Attention Optical Tensiometers) at room temperature. Deionised water (0.4 μL) was automatically dropped on the coating surfaces, and Laplace–Young fitting was applied on contact angle measurements. The measurements were repeated 5 times at different positions of the multifunctional coating for an average value.

## 3. Results and Discussion

### 3.1. Synthesis Ag NP, Si QD, and Perfluorosilane Precursors and Blank Hybrid Coating

**Figure 2:**
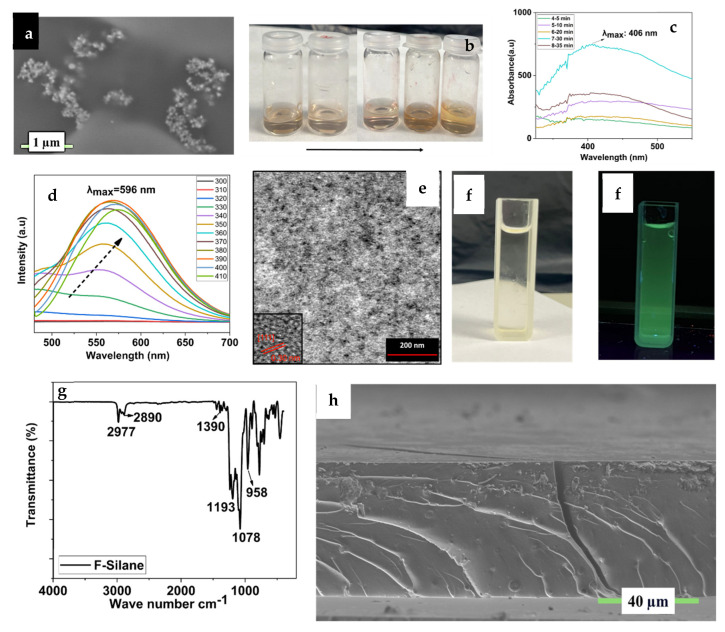
(**a**) SEM image of the synthesised Ag nanoparticles; (**b**) daylight pictures of the time-dependent formation of the Ag nanoparticles; (**c**) SPR spectra of the synthesised Ag nanoparticles; (**d**) PL spectra of the synthesised Si QDs; (**e**) TEM and HR-TEM images of the synthesised Si QDs; (**f**) images of the Si QDs under daylight and UV light; (**g**) FT-IR of the prehydrolised FAS compounds; (**h**) cross-sectional image of the blank hybrid coating.

Since metal nanoparticles show exciting features due to their different shapes, sizes, and compositions, it is necessary to know that metallic nanomaterials are significantly linked to their physical, chemical, and optical properties [17,18,19,20], and, among metal nanoparticles, silver (Ag NPs) peaked due to their physical, chemical, and biological characteristics mainly originating from the size. Also, shape, composition, and crystallinity are important in crystalline AgNPs compared to their bulk forms. Many efforts are widely known for Ag NP in anti-bacterial and anti-cancer therapeutics, diagnostics, and optoelectronics. Surface plasmon resonance (SPR) absorption was characterised and found to be 400 nm. Also, the size, shape, and morphological characteristics of Ag NPs were investigated using SEM. Therefore, results showed that as-prepared Ag NPs were mostly monodispersed (Figure 2a), spherically shaped, and homogeneously distributed, with a size of 100 nm. Daylight solutions of these obtained Ag NPs are shown in Figure 2b. The brownish/yellow solution proves the formation of Ag nanoparticles. Aliquots of the particle formation for 5, 10, 20, 30, and 35 min show the surface plasmon resonance (SPR) absorption, which was evidenced by UV-Vis spectroscopy (Figure 2c). Figure S1 presents a histogram of Ag NP with an average diameter of 61 ± 7 nm (Figure S1) obtained from the SEM images. Results clearly showed that silver nanoparticles were prepared by the Turkevich method, and citrate ligands protected the surface of the nanoparticles due to the nature of the synthesis [21].

The reaction between APTES and sodium ascorbate in aqueous solution was carefully performed in Si QD synthesis under UV light. A sketch of the reaction is already proposed in Figure 1. After the synthesis reaction, the Si QD’s growth and composition can be observed by PL, TEM, HR-TEM, and STEM (Figure 2d–f). Due to the protected and controlled surface of the Si QD, a monodispersed particle distribution with a petite nanocrystal size (~2 nm) with a spherical shape was detected, as imaged by STEM and HR-TEM (Figure 2e inset). The HRTEM image also unveils the high crystallinity of the Si QD and lattice fringes with 0.30 nm interplanar spacing, consistent with the (111) plane of diamond. Si was detected under visible light (Figure 2f); the yellowish solution showed bright green–cyan emission under UV light (Figure 2f). Visual examination of the Si QD solution under visible and UV light unveiled a bright green–cyan emission, which can be used for different material applications. Visible light emission of the Si QD arises from quantum confinement due to the size of the Si nanostructures. It is very prominent that silicon exhibiting a weak PL and a long exciton-hole recombination time transforms it into a material that is hard to prepare in massive amounts with monodispersity and uniformity. Si QDs showed visible emission between 520 and 590 nm (Figure 2d) with different excitation wavelengths between 300 and 410 nm, respectively. Results showed that emission maxima corresponding to the λ_ex_ = 596 nm are decreasing constantly, and this can be attributed to the QD emission peaks changing formation and growth steps with varying surface environments. The excitation wavelength was increased by 10 nm at every additional experiment [22,23].

To optimise the surface characteristics of the synthesised multifunctional materials, a special coupling agent, F 8261 (FAS), was utilised as a surface modifier to treat the surface of the multifunctional coating in this study. After the acidic hydrolysis and condensation, which is shown in Figure 2g, after at least 1 day, carbon and fluoride bonds (-CF_2_-CF_2_-) will be transferred from FAS to the surface of the multifunctional coating during the synthesis procedure. Therefore, the final material will present strong hydrophobicity and self-cleaning features. After the hydrolysis and condensation reactions, Si-O bonds are seen at 1078 cm^−1^ and C-F bonds are at 1012 cm^−1^, together with 2977–2980 cm^−1^ bonds, which are still ongoing hydrolysable alkoxy groups [24,25,26,27,28].

For the hybrid blank nanocomposite coating, 3-glycidyloxypropyl trimethoxy silane—GLYMO—was used as a matrix, and this epoxysilane was reacted with aluminium tri-seconder butoxide Al(O_s_Bu)_3_. Al(O_s_Bu)_3_ was also stabilised and chelated with ethyl acetoacetate and called the aluminium complex. Chelation was conducted to decrease the air reactivity of Al(O_s_Bu)_3_. After the hydrolysis and condensation reaction of GLYMO, the aluminium complex was added dropwise while the stirring procedure was performed. Coating solutions were applied to aluminium substrates (Assan Aluminium Industry, alloy 1050, 0.8 mm Al plate, 10 × 10 cm) by the spray coating method, and a chemical and physical investigation of this blank coating was performed. SEM investigation in Figure 2h clearly shows the coating’s hybrid material behaviour and surface morphology. Because of the high conductivity of the aluminium substrate, coating properties were investigated using carbon-coated glass plates. Cross-sectional SEM pictures of the coating show a very compact chemical structure with almost no porosity and no shrinkage of the sol-gel network. Also, the uniformity’s complex chemical structure and very homogeneous distribution can be seen.

### 3.2. Synthesis and Step-by-Step Investigation of the Multifunctional Hybrid Coating

**Figure 3:**
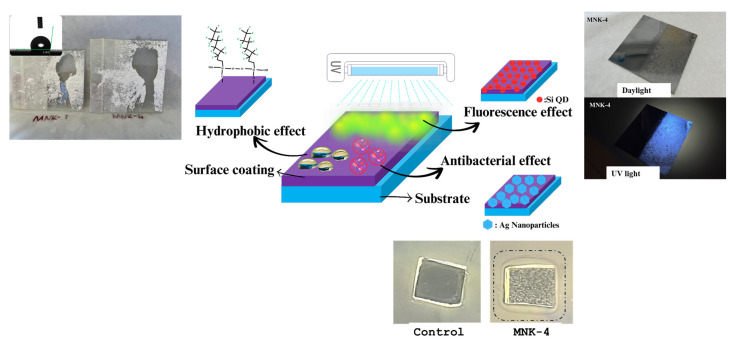
Graphical representation of the total synthesis of the blue fluorescence light-emitting, antibacterial, and hydrophobic self-cleaning multifunctional coating.

For this study, an inorganic–organic hybrid structure was selected as the matrix component during the synthesis of the multifunctional coating procedure. All the smart items of multifunctionality were introduced stepwise, like Si QDs, then prehydrolised fluorosilane compound, and finally, Ag NPs were introduced. In general, alkyltrialkoxysilanes (R-(Si-OR′)_3_, where R = alkyl mercapto, alkyl glycidyl, long alkyl, or acryl, and R′ = alkoxy groups) are promising precursors for coating materials during the synthesis of modified surface coatings, and therefore the chemical and dimensional features of this sol-gel reaction were taken as the template procedure [29,30,31]. There are many advantages of using this matrix since (i) its beginning reactions contain the semi-hydrolysed alkoxide precursor and allow the formation of –OH groups; (ii) epoxy branched alkoxide reactions form the connected and possibly elongated clusters, which is proper for the molecular control; and (iii) the epoxysilane precursor curing procedure is well known, compatible functionalities may be appropriately added, and final curing can be performed (Figure 3).

**Figure 4:**
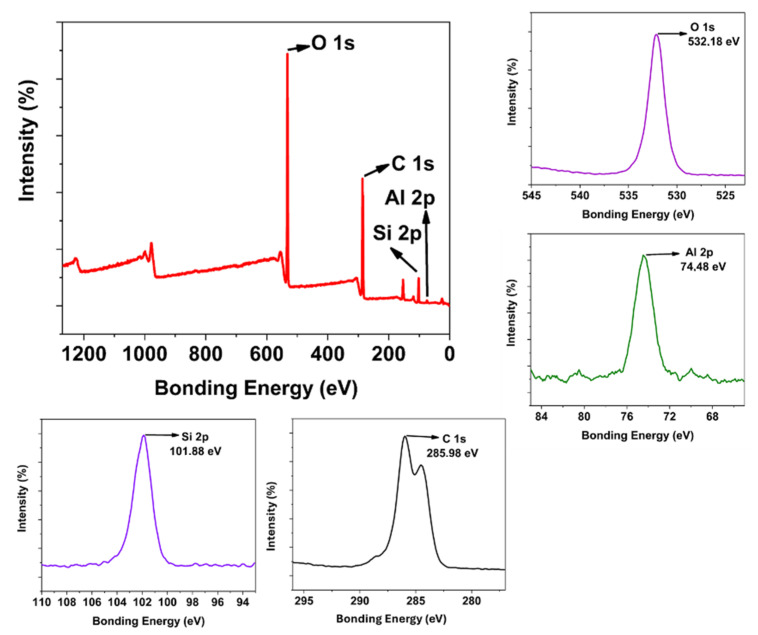
Survey and HR-XPS analysis of the blank MNK-1 coating.

Starting from the blank matrix coating, every functional feature was added separately, and the obtained surface coating was analysed extensively. Figure 3 shows the image representation of the multifunctional coating procedure. The final multifunctional coating shows the self-cleaning hydrophobic effect, visible range fluorescence emission, and antibacterial feature. Figure 4 shows the XPS and HR-XPS characterisation of the blank coating. In the survey spectrum, C 1s, O 1s, Al 2p, and Si 2p peaks are seen, proving the engineered structure of the coating. Single Al 2p (74.48 eV) and Si 2p (101.88 eV) peak positions are compatible with one Si and Al binding type in the coating formulation. Deconvoluted O 1s peaks show three peaks exhibiting the three different types of oxygen atoms for Si-O, Al-O, and O-C seen at 533 eV, 534 eV, and 535 eV, respectively [32,33,34,35]. Also, C 1s peaks detected at 288.1 eV, 285.98 eV, and 284.4 eV show its multibonding nature. TG-DTA, XRD, and FT-IR (Figure S2) results of the cured MNK-1 coating reveal the general information about the thermal, crystalline, and surface background of the MNK-1 coating. Analyses were conducted in a nitrogen environment from 0 to 900 °C. In Figure S2, TGA and DTA spectra are seen. A significant weight loss in two steps was detected starting from the beginning. Below 250 °C, corresponding 8% weight loss is considered the evaporation of water and other volatile components. Until 350 °C, probably all other organic molecules were removed, and close to 35% weight loss between 350 and 550 °C can be attributed to the further condensation of polymeric networks. This process continues until 800 °C with a 12% weight loss. Therefore, two exothermic peaks can be detected in DTA spectra. A total 55% weight loss for the MNK-1 was detected. An XRD peak (Figure S2) at 2θ = 21.68 appears, representing the bulk SiO_2_ formation. This peak can be anticipated since the whole system is a template of aluminosilicate structure based on the sol-gel condensation. FT-IR peaks at 1050 cm^−1^ appear for Si-O bonding, and the O-H peak is seen at 3385 cm^−1^, and the C-H peaks at around 2865–3000 cm^−1^ unveil the sol-gel-based hybrid chemical structure of the coating (Figure S2). The obtained surface coating shows a low water contact angle, as seen in Figure S2, confirming the polar character of the obtained surface.

**Figure 5:**
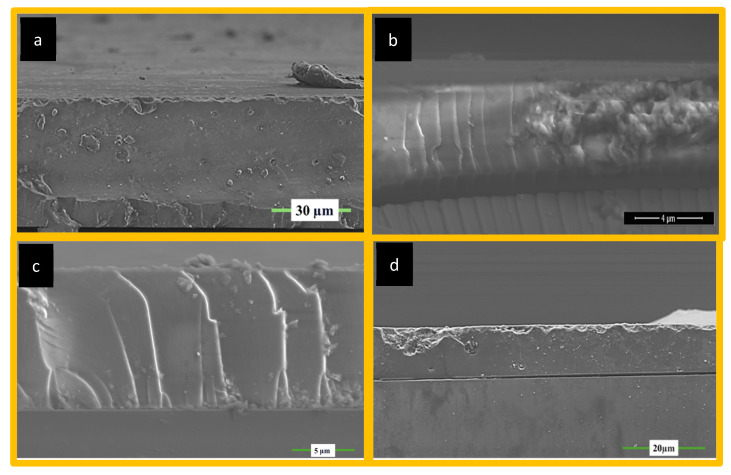
(**a**) Blank coating; (**b**) Si QD-embedded viable emitting coating; (**c**) Si QD- and Ag NP-introduced visible emitting and antibacterial coating; (**d**) Si QD- and Ag NP-introduced, and FAS-modified visible light emitting, antibacterial, and hydrophobic self-cleaning multifunctional coating.

**Figure 6:**
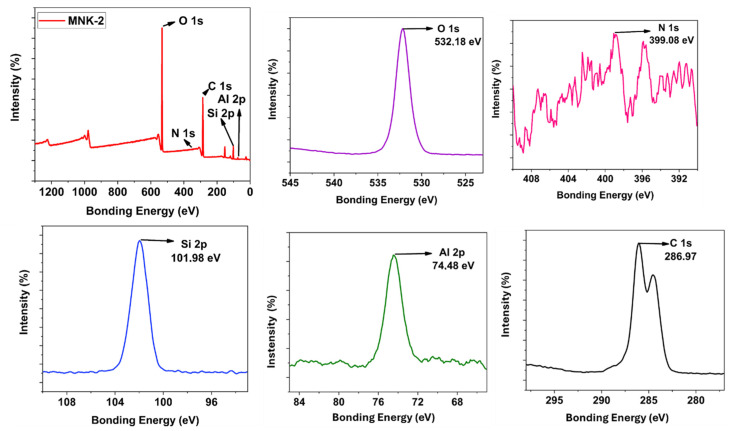
Survey and HR-XPS of the Si QD containing the MNK-2 coating.

Figure 5 shows the SEM images of all surface coatings starting from the blank (MNK-1), Si QD-embedded (MNK-2), Si QD-embedded, and FAS-modified (MNK-3). Finally, Si QD was embedded, FAS was modified, and Ag NP was decorated (MNK-4) with a multifunctional coating, and glass substrates were used. Necessary preparation was performed prior to measurement before collecting the SEM images. Thicknesses vary from 5 to 15 μm, while the blank coating is around 40 μm. Cross-sectional measurements clearly show the compact and homogeneous coating material character, where no cracks or holes are observed. Adding further functionalities does not change the leading chemical and physical structure of the functional coatings on the glass substrates. In the SEM image of MNK-2, Si QDs are not perfectly visible due to their tiny size in the cross-sectional image. Still, when the XPS analysis was investigated deeply (Figure 4 and Figure 6), all the necessary atoms were visible in the survey XPS after the Si QD embedding. The surface of this new coating shows a slightly smaller contact angle with water droplets, possibly due to the hydrophilicity of the Si QDs and their surface modification with alkylamine side groups (Figure S2). XRD analysis, survey XPS comparison with blank coating (MNK-1), TG-DTA analysis, and water contact angle of the MNK-2 coating are presented in Figure S3. XRD diffraction spectra show a similar bulk SiO_2_ behaviour with a shifted 2θ = 22.6 peak (Figure S3). Comparison of the survey XPS spectra of MNK-1 and MNK-2 reveals that the only difference between the two spectra is the nitrogen peak, which appears very slightly at around 399.08 eV (Figure S3). This is due to the nitrogen atom of the Si QD precursor. The same thermal conditions gave the parallel weight losses in two main steps (with the beginning volatile compounds), which were detected as 57% total (Figure S3). This is compatible with introducing the Si QDs into the coating structure. Almost the exact decompositions, as seen in MNK-1, are observable. Contact angle (70 degrees) is also predictable since the hydrophilic quantum dot nature does not vary the water contact angle of the surface coating (Figure S3).

In the new MKN-3 coatings, fluorosilane modification seems compatible with the Si QD-embedded new coating and does not have any adverse effect on the old coating. Still, no cracks and no porosity were observed with perfluor modification. The SEM image shows the compact and solid character of the coating after perfluor modification. As presented in Figure S4, the addition of second functionality (self-cleaning by FAS) provided a 113-degree water contact angle (Figure S4), and the fluor amount can be detected by XPS and EDX analysis. TG-DTA (Figure S4) analysis of the same MNK-3 coating unveils the same thermal character, which was decomposed in 3 steps as observed before. In the end, a total of 55% weight loss was observed. The FT-IR spectrum (Figure S4) provides the Si-O and C-H character together with the CF_2_ peaks of the perfluor modification. The XRD diffractogram shows a single peak at 2θ = 20.8 degrees (Figure S4), proving that even perfluor modification does not change the crystallinity of the hybrid coating. EDX atomic mapping for MNK-3 coating shows the fluor atom in a homogeneously distributed perfluor group throughout the coating (Figure S5). Coatings MNK-2, MNK-3, and MNK-4 are almost perfectly compatible with each other, and as mentioned, the addition of a new smart functional character can be observed by the XPS and EDX atomic mapping investigation (Figure S5). Since Si QDs, perfluoro alkyl silane, and Ag NP can be added instantaneously, GLYMO acts as a perfect matrix material for the multifunctional coating [36,37].

Figure 5 represents the hybrid coating modified with Si QDs, which selectively provides the visible region emission with the help of these Si QDs. When analysed, the MNK-2 nanocomposite shows clear survey peaks for Al, O, Si, and C atoms. Additionally, when the HR XPS peaks for this coating are examined in detail, a single peak at 74.48 eV for Al and another at 101.98 eV for Si atoms are observed. This indicates that the Si QD structure is primarily embedded in the nanocomposite body rather than positioned closer to the surface. It is understandable that otherwise, a different Si HR XPS peak could have been observed for Si QD in the HR XPS.

**Figure 7:**
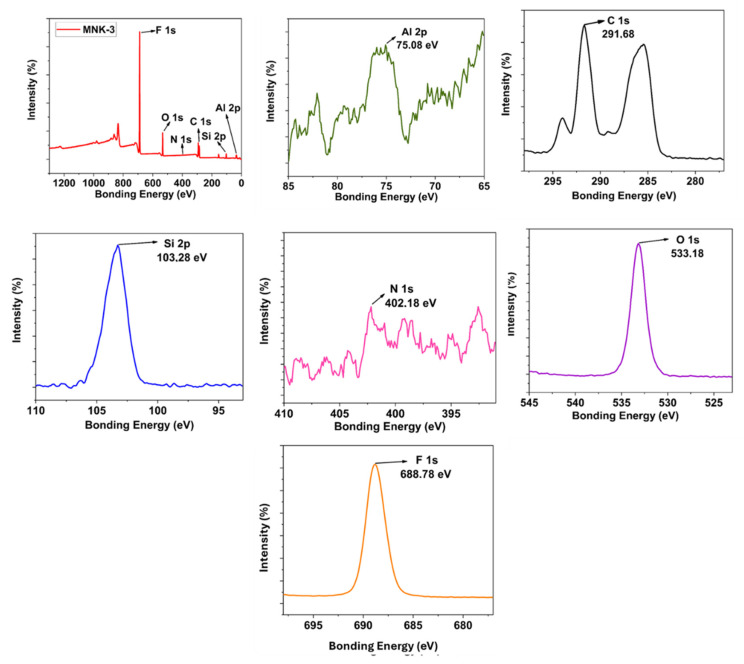
Survey and HR-XPS investigation of the MNK-3 coating.

The same observation can be speculated for the N atom peak of the Si QD starting material AMEO (amino propyl triethoxysilane). Although certain of its presence, the N peak is observed as a low-intensity and broad peak in the 399–396 eV range. Although the high-resolution XPS (HR XPS) peak for the oxygen atom is observed as a single peak at 532.18 eV, a triple peak with different intensities indicates the expected diversity for the carbon atom. Thus, the atomic analysis of the embedded hybrid coating with Si QD is consistent with the formulation. When this coating (MNK-2) is compared with both Si QD and the FAS-modified coating (MNK-3), a prominent F peak is noticeable in the survey XPS for MNK-3 (Figure 7). This peak, observed at 688.68 eV, confirms that the FAS structure is homogeneously distributed on the surface due to the hydrophobic effects. As expected, the N atom peak from Si QD is again observed as a broad and low-intensity peak in the 402–394 eV range. Interestingly, the HR XPS peak is detected at a very low intensity for the Al atom at 75.08 eV when compared to MNK-2. The FAS modification significantly modifies the XPS peaks of Al atoms in the corresponding MNK-3 coating. When the Si HR XPS peak in MNK-3 is examined, a secondary peak around 104 eV is observed in addition to the expected central Si peak (103.28 eV). This is likely due to a shift originating from the Si atom in the FAS molecule. The Oxygen HR XPS peak in MNK-3 shifts to 533.18 eV and is again observed as a single peak. For carbon atoms, approximately five different carbon deconvolutions are observed, originating from different carbon characters in the main polymer structure and perfluor groups. Peaks for carbon atoms based on perfluorosilane are observed in the 291–294 eV range. Thus, it has been demonstrated that the basic FAS modification causes shifts in electron density and peak positions in all aluminium, nitrogen, oxygen, carbon, and silicon atoms.

**Figure 8:**
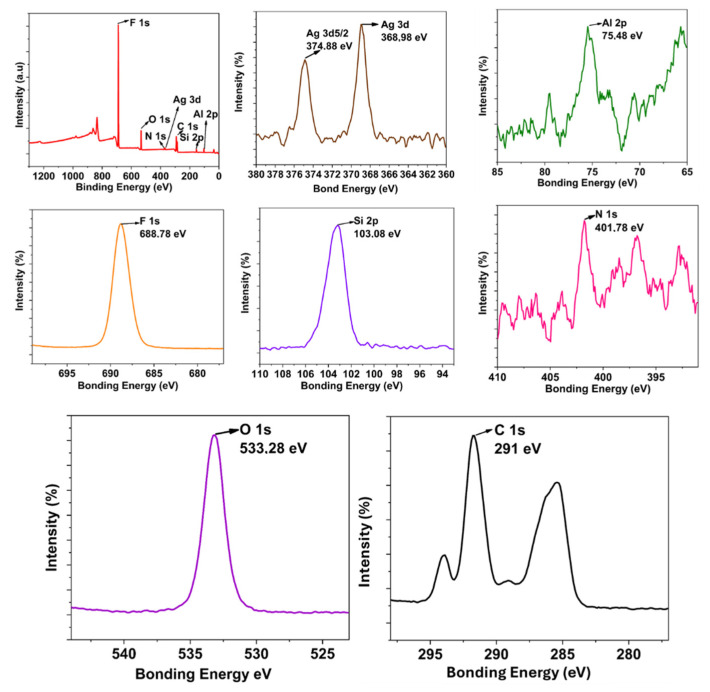
Survey and HR-XPS investigation of the multifunctional MNK-4 coating.

Additionally, the modification is evident in the fluorine peak, indicating a hydrophobic character. As a final multifunctional coating (MNK-4), when the XPS and HR XPS analysis of the Ag nanoparticle, FAS, and Si QD-modified nanocomposite is examined (Figure 8), the most significant difference was observed to belong to the Ag nanoparticles. The 3d 5/2 and 3d 3/2 peaks belonging to Ag nanoparticles are observed sequentially at 374.88 eV and 368.98 eV, respectively. Literature analysis shows that Ag nanoparticles exhibit 3d peaks close to these values. This has been added as the final functionality providing antibacterial properties after Si QD and FAS molecule modification, resulting in a multifunctional hybrid coating [38]. All other HR-XPS peaks for MNK-4 are similar to those of MNK-3. All existing atomic structures exhibit the same characteristic peaks as MNK-2, consisting of C, O, Si, Al, F, or N. This indicates that the coating with added Ag nanoparticles is consistent with the expected XPS analysis. Thermal analysis showed a total 52% weight loss in three steps, and XRD diffraction resulted in 2Q 19.08 degrees, which shows a noticeable shift when compared to MNK-1, MNK-2, and MNK-3. The FT-IR spectrum shows similar peaks to the MNK-3; no observable peaks for Ag nanoparticles were observed. The water contact angle shows 119°, proving the continuation of the self-cleaning feature of the multifunctional hybrid coating (Figure S6). While the current study did not include a full life-cycle or ecotoxicological analysis of the materials obtained and used, used Ag NPs were selected in our study due to their well-documented and effective antibacterial properties, which allow for lower required dosages compared to other biocides. In our synthesis, the amount of Ag NPs used was minimal (1 wt% of the final material), and efforts were made to immobilise them within the matrix, reducing the risk of leaching and environmental release. 

### 3.3. Material Applications of the Multifunctional Hybrid Coating

For the scratch and adhesion properties of the multifunctional materials, complete tests were performed according to the cross-cut/tape test of the ASTM D 3359. According to the applied test, huge surface cracks or relatively high amounts of removed material from the surface may be attributed to the weakness of the adhesion. As given in Figure S7, the tests indicated that coatings have a 5B value as the test result for the scratch resistance. Observation showed that the tape test removed no disintegrations, excessive lines, or coating material. The adhesion of the coatings on aluminium substrates was also determined. All coatings’ GT/TT ratio was excellent, which means it was equal to zero. According to the test, it is already known that if adhesion is excellent, GT/TT = 0; if there is no adhesion between substrate and coating material, GT/TT = 5. According to these tests, coatings adhere to Al surfaces, which shows a peaking behaviour for the obtained multifunctional coatings. Antibacterial, self-cleaning, fluorescent, and multifunctional coatings can be applied safely onto daily life materials; for example, metal parts of the kitchen components [39,40,41].

In addition to these promising physical features, widely known phenomena and experimental design were tested in this study, where a water droplet slides down a tilted substrate surface. Here, the multifunctional hybrid coating was treated with an arranged design. It was anticipated that, due to the significantly reduced surface tension between water and the hybrid coating, the applied water droplet would slide down without any residue of the droplet adhering to the hybrid coating surface. Applied water droplets play the cleaning role, dragging the dirt particles away from the surface. Compared to the unmodified nonhydrophobic surface, the hybrid multifunctional coating shows the water droplet sliding down the multifunctional platform without leaving a water trail and cleaning the dirty surface.

**Figure 9:**
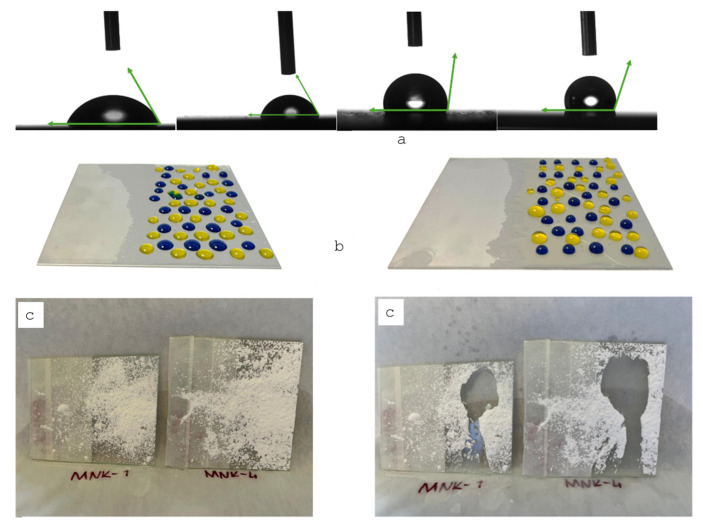
(**a**) Average water contact angles of MNK-1 (60–65 up left), MNK-2 (70–75 up second left), MNK-3 (110–115 up third left), and MNK-4 (115–120 up right); (**b**) water droplets on the MNK-1 (middle left) and MNK-4 (middle right); (**c**) self-cleaning analysis before (left) and after (right) for MNK-1 and MNK-4.

**Figure 10:**
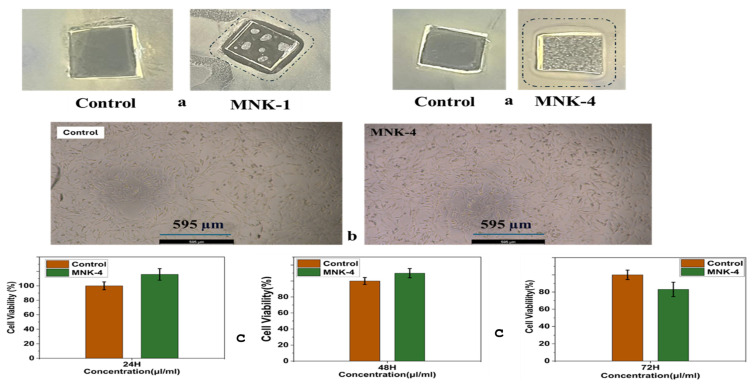
(**a**) Antibacterial test of the MNK-1 and MNK-4, together with the control coating; (**b**) cell viability of control and after MNK-4 treatment; (**c**) images of cell viability graphs after 24 h, 48 h, and 72 h.

We also observed that adhesion between the dust particles and the multifunctional coating is reduced, so the movement of a water droplet easily washes away the dirt particles. Also, Figure 9 shows the water contact angles changing from 60–65 to 115–120 degrees of the coatings by adding each component starting from the MNK-1 coating and the MNK-4 multifunctional coating. The visual exhibition on the coatings with yellow and blue droplets (Figure 9b) reflects the increased contact angle of the water. Also, the images of the self-cleaning experiments before and after the dirt removal (Figure 9c). Hence, it is clearly shown that FAS-treated multifunctional coating has the force of water to remove the dust and dirt, gravitationally pushing the dirt to the bottom of the multifunctional coatings, which keeps the surface clean and transforms the coating into a self-cleaning coating [42,43]. This feature is one of the most helpful for the people who desire easy-to-clean and self-cleaning surfaces in daily applications, which can be obtained by this kind of multifunctional material. For the antibacterial test of the MNK-4 coating, *E. coli* and *S. aureus* bacteria were incubated in agar with the glass coating samples. Results clearly showed that (Figure 10a,b) the inhibition rate of the MNK-4 multifunctional coating was higher than that of the control. Also, MNK-1, which contains no Ag nanoparticles, showed almost no inhibition against the bacteria. The results indicate that the MNK-4 multifunctional coating has a robust inhibition rate against *E. coli*, with an average of 18.69 mm. MNK-1 and control samples have much lower inhibition rates, with averages of 1.96 mm and 1.74 mm, respectively (Figure 10a). These findings suggest that the multifunctional MNK-4 coating has the highest efficiency compared to the MNK-1 and control coatings in preventing the growth of *E. coli*. It is pretty easy to attribute a higher inhibition rate of the multifunctional coatings to the Ag nanoparticles, which behave as a selective antibacterial agent on the surface [44,45,46]. Due to the unique silver ion-releasing feature of the multifunctional MNK-4 coating, it can be used as an industrial coating in kitchens or similar environments, even on metal or glass surfaces. In addition to the silver ions, chemical stability and an additional hydrophobic effect can prevent bacterial growth since water contact angles showed that the multifunctional MNK-4 coating has a higher contact angle than the previous ones. Due to the hydrophobic surface, reduced bacterial adhesion, promoting self-cleaning properties, may have been observed. Since the control and MNK-1 have no special treatment against bacterial adhesion, they become more susceptible to bacterial adhesion and growth. Results showed that, in addition to the self-cleaning and high hydrophobic water contact angle results, multifunctional coating MNK-4 has an antibacterial feature against hazardous *E. coli* bacteria. Cell viability results showed a high compatibility with the beginning cells, and graphical representation proved the cell resistance against population decrease (Figure 10b,c).

**Figure 11:**
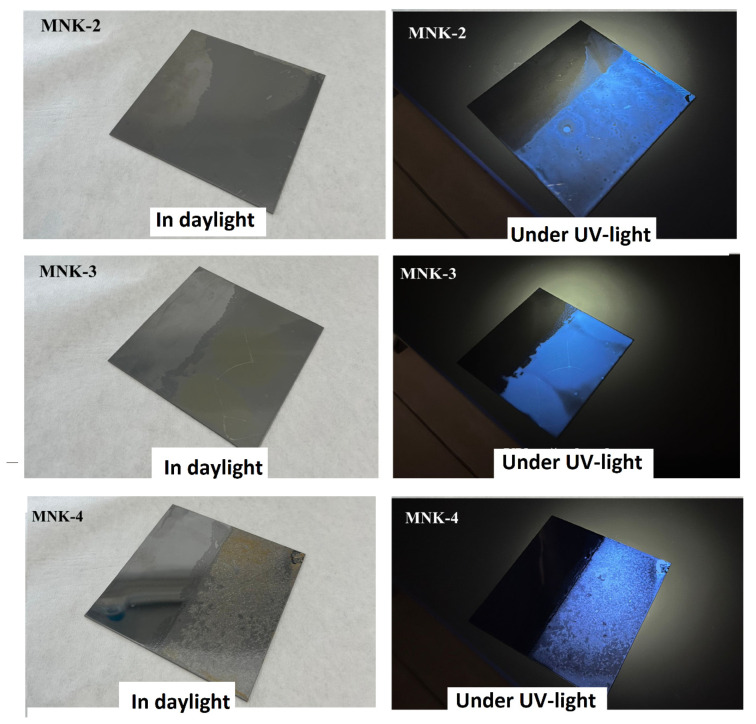
Fluorescent feature of the Si QD-embedded MNK-2, MNK-3, and MNK-4 multifunctional coatings.

Si QD-supported fluorescence properties of the MNK-2, MNK-3, and MNK-4 multifunctional coatings are shown in Figure 11. Since Si QD embedding distinctively leads to the fluorescence features, all the coatings emit cyan-blue fluorescence. It is widely known that nanocomposite hybrid coatings, or materials that combine fluorescence and biocompatibility, may have huge roles in biological systems. Quantum dots provide exciting opportunities, such as biological imaging, sensing, drug delivery, and a visible light-emitting platform for biological systems [47,48,49]. Since tunability in the emission wavelength of quantum dot embedded systems enables real-time monitoring, targeted therapy is possible. Also, fingerprint detection with fluorescent coatings or materials has become widely known in daily life. As a part of multifunctional material, fluorescence emission features have a promising future for theranostic, biological, and material applications. Since Si QDs are very resistant to pH changes and their emission feature is strongly resistant and stable against environmental factors, one may apply these multifunctional materials on different surfaces such as ceramic, glass, metal, or wood substrates. As also shown, the emission properties of the coating were not affected by the step-by-step construction of the multifunctional material. Since cytotoxicity results are acceptable, multifunctional materials have massive potential for future applications [50,51,52]. The methodology employed in this study deliberately utilised widely available and low-cost precursors like APTES, AgNO_3_, solvents, GLYMO, Al alkoxides, and FAS in mild conditions, avoiding expensive or hazardous ligands (e.g., trioctylphosphine) and rare metals like cadmium. This makes the process inherently cost-effective when compared to traditional high-temperature or toxic precursor-based material synthesis. Furthermore, the water-based or alcohol-based solvent systems and ambient pressure processing enable facile scale-up with minimal energy demands, which further supports economic sustainability. Developing multifunctional smart materials exhibiting different features is expectedly promised to present significant challenges due to available chemical and physical incompatibilities among these properties. For instance, in this study, the effectiveness of antibacterial metal ions often increases in hydrophilic environments, whereas hydrophobic surfaces may reduce their efficacy. Similarly, fluorescent agents may degrade under exposure to light and oxygen, compromising long-term stability. These interactions complicate the formulation process and the overall performance of the final material. Also, such complex systems typically involve multi-step procedures and tightly controlled conditions, making scalability and cost-efficiency difficult to achieve very easily. Therefore, successfully implementing multifunctional smart materials that provide multifunctionality features for new applications requires simultaneous optimisation of structural compatibility, environmental safety, production feasibility, and economic viability.

## 4. Conclusions

In this study, the step-by-step production of a hybrid coating material with significantly different properties related to the multifunctional materials was examined in detail. A hybrid innovative material was produced in the form of a coating, which emits light in the visible spectrum due to the addition of Si QD, exhibits antibacterial properties against *E. coli* and *S. aureus* due to Ag NP, has no side effects on human health according to the cytotoxicity tests, and is self-cleaning due to perfluorination. The sol-gel reaction, controlled by hydrolysis and condensation reactions, played a key role in producing this hybrid multifunctional material and provided a suitable platform for the seamless implementation of the step-by-step functionalisation procedure. As a result, a coating formulation that simultaneously exhibits antibacterial properties, emits fluorescence, and shows a self-cleaning effect was obtained as a part of the multifunctional nanomaterial concept.

## Data Availability

The original contributions presented in this study are included in the article/Supplementary Materials. Further enquiries can be directed to the corresponding author(s).

## Supplementary Materials

The following supporting information can be downloaded at: https://www.mdpi.com/article/10.3390/polym17152139/s1, Figure S1. Particle size distribution of the obtained Ag nanoparticles; Figure S2. TG-DTA, XRD, FT-IR, and water contact angle investigation of the cured blank MNK-1 coating; Figure S3. XRD spectrum, MNK-1 and MNK-2 curves XPS comparison, TG-DTA investigation, and water contact angle image of the Si QD-embedded MNK-2 coating. Figure S4. TG-DTA investigation, FT-IR spectrum, and XRD diffraction with the water contact angle of the MNK-3 coating. Figure S5. EDX atomic mapping for C, Al, O, Si, F, and N for the MNK-3 coating. Figure S6. TG-DTA investigation, FT-IR spectrum, and XRD diffraction results with the water contact angle of the MNK-4 coating. Figure S7. Cross-cut test of the MNK-1, MNK-2, MNK-3, and MNK-4 coatings.
