# Supplementary material for: Multifunctional, Biocompatible Hybrid Surface Coatings Combining Antibacterial, Hydrophobic and Fluorescent Applications
Source: Polymers (Basel). 2025 Aug 5;17(15):2139. doi: 10.3390/polym17152139 (PMC12349159; doi:10.3390/polym17152139)
Supplement: Supplementary file 1 [file polymers-17-02139-s001.zip › polymers-3783019-supplementary.pdf]

Supporting Info

Multifunctional, Biocompatible Hybrid Surface Coatings Combining Antibacterial, Superhydrophobic and Fluorescent Applications

Gökçe ASAN, Osman ARSLAN

Corresponding Author: o.arslan@izu.edu.tr

Istanbul Sabahattin Zaim University, Food Engineering Department,34303, ISTANBUL

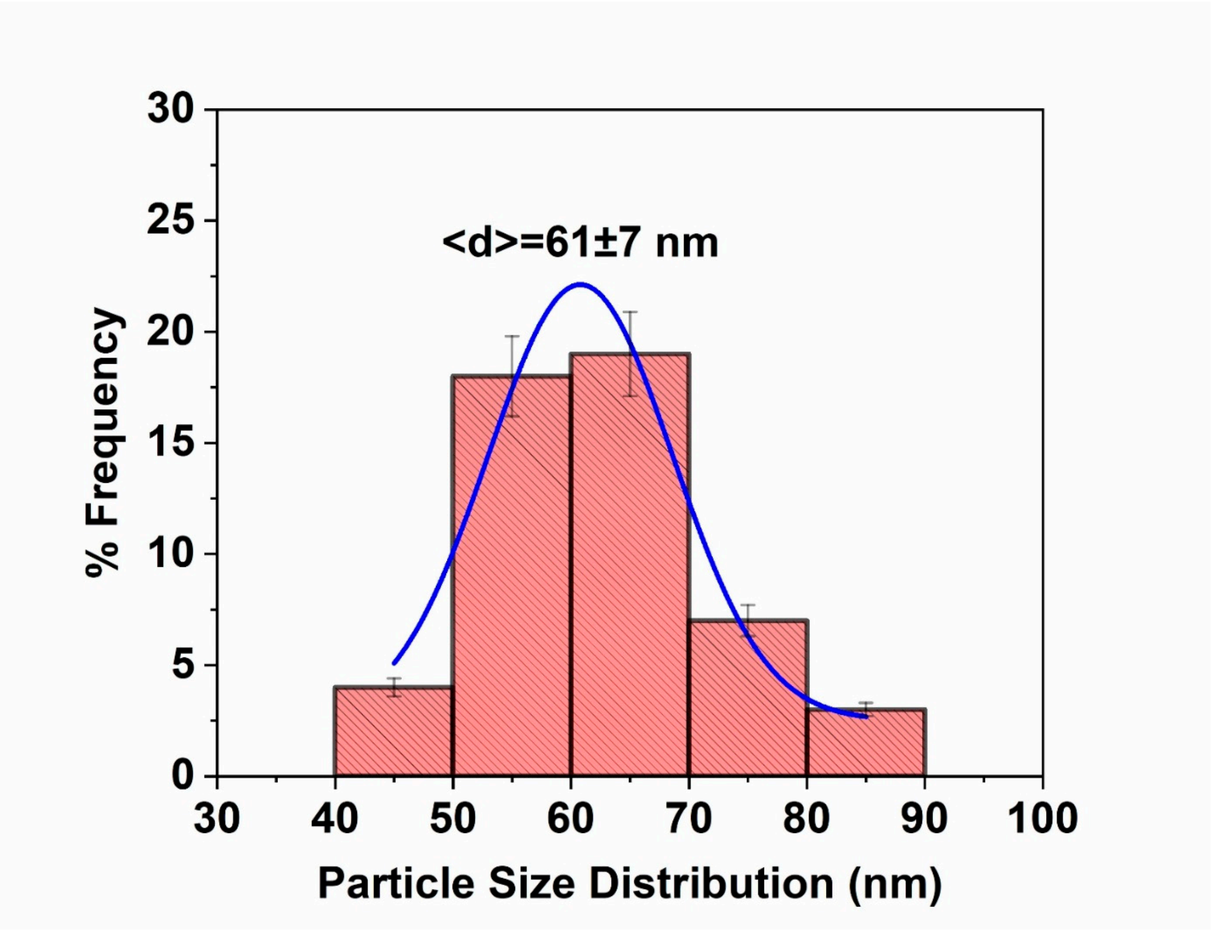

Figure S1: Particle size distribution of the obtained Ag nanoparticles

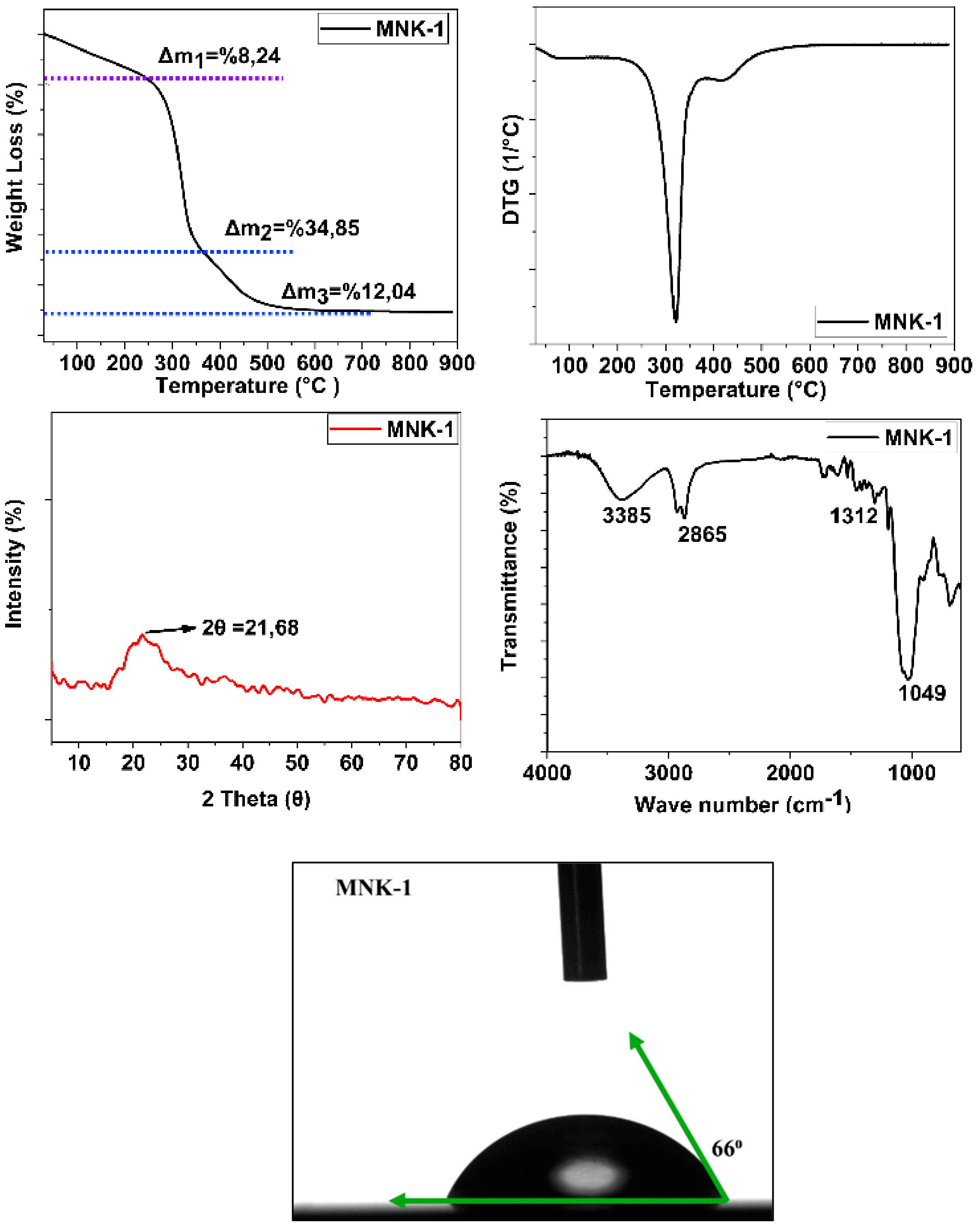

Figure S2: TG-DTA, XRD, FT-IR and water contact angle investigation of the cured blank MNK-1 coating

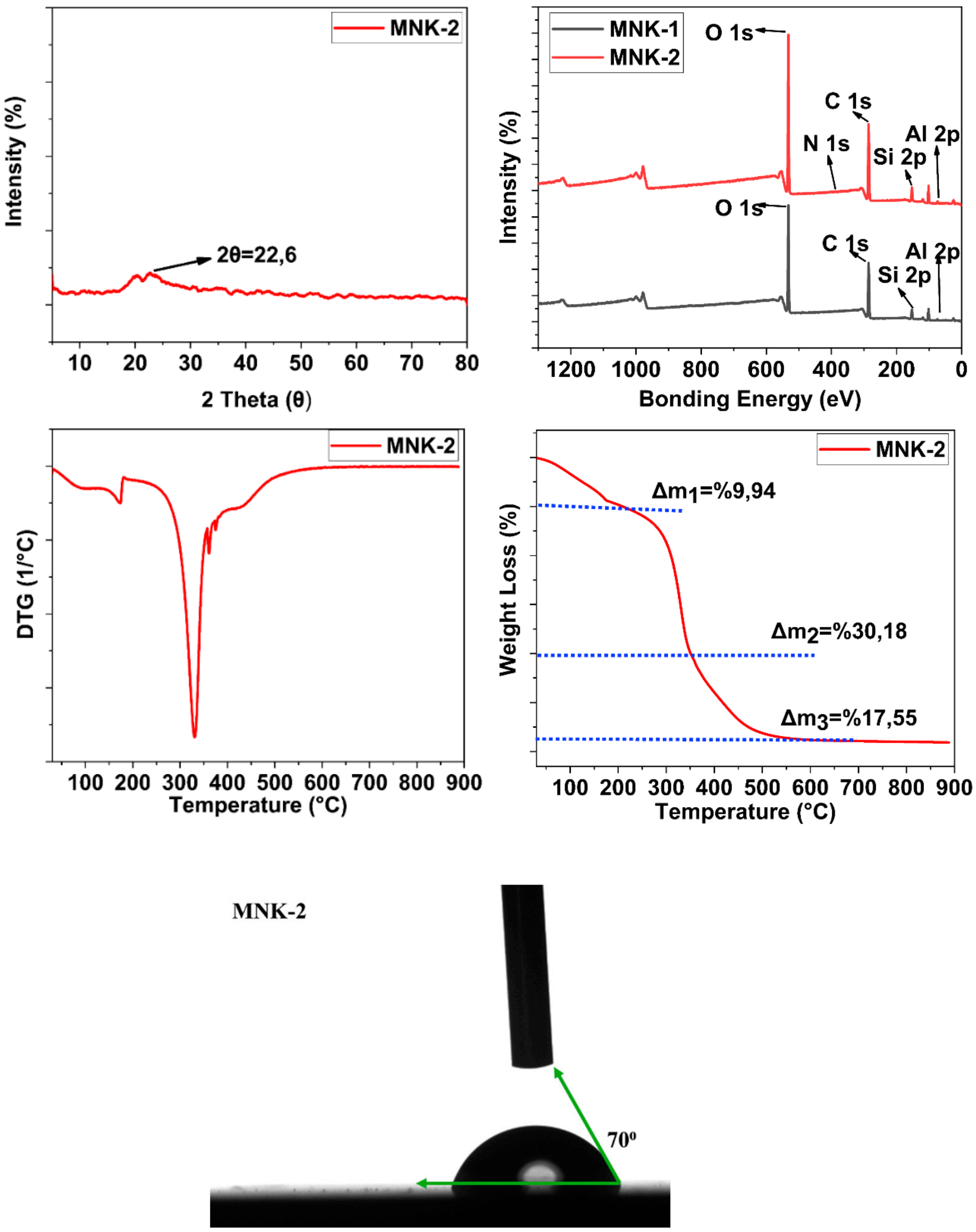

Figure S3: XRD spectrum, MNK-1 and MNK-2 surves XPS comparison, TG-DTA investigation and water contact angle image of the Si QD embedded MNK-2 coating

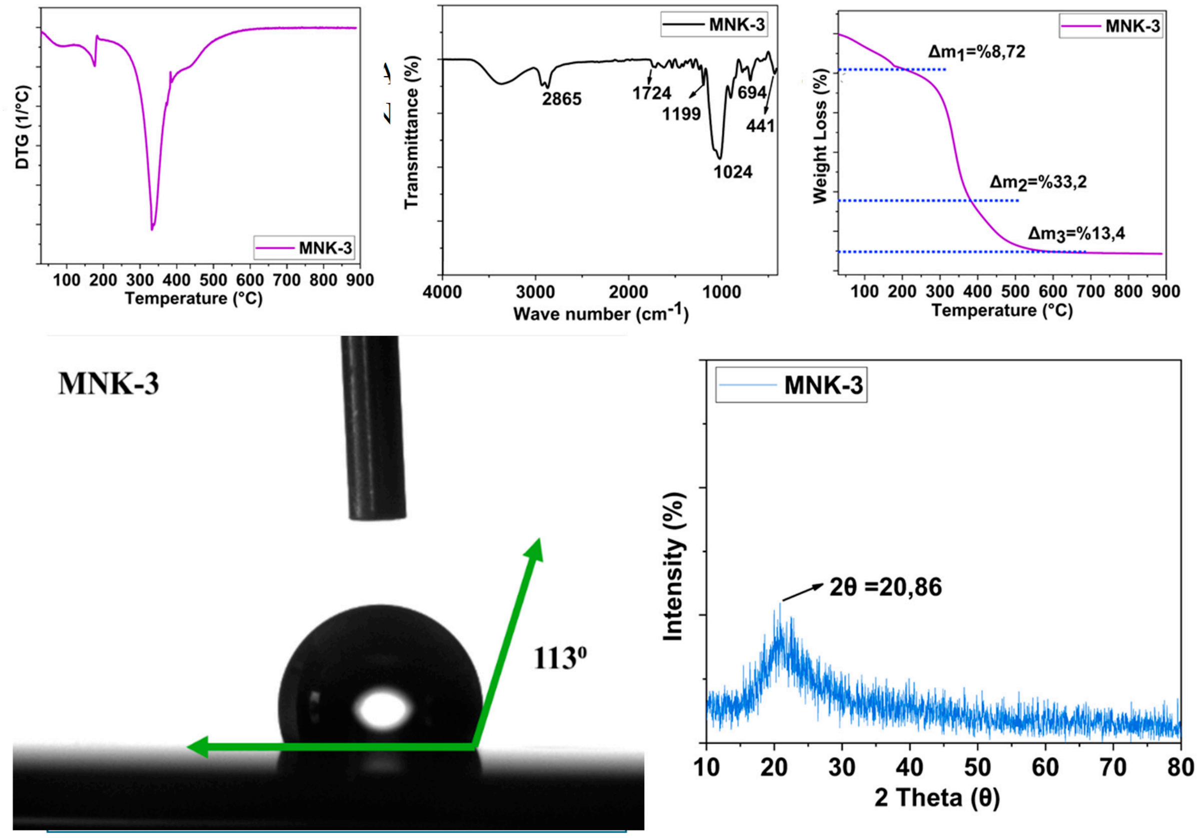

Figure S4: TG-DTA investigation, FT-IR spectrum, XRD diffraction and water contact angle image of the MNK-3 coating

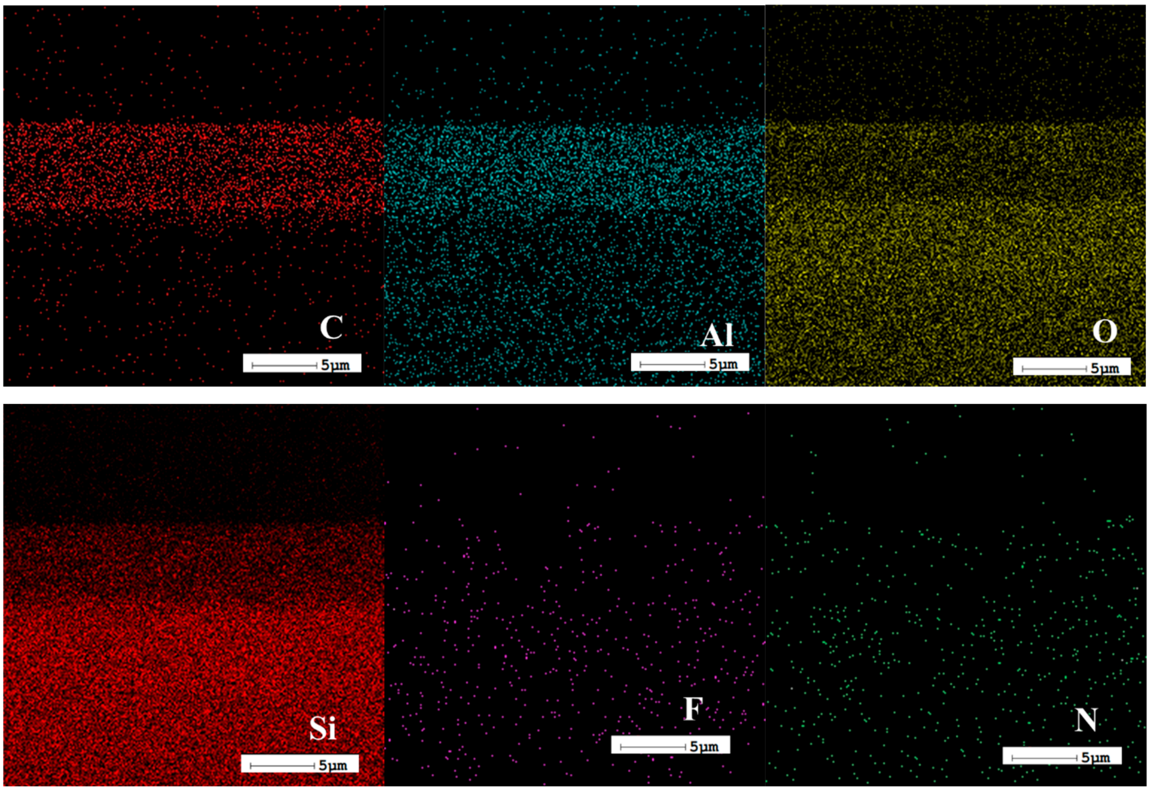

Figure S5: EDX atomic mapping for C, Al, O, Si, F and N for MNK-3 coating

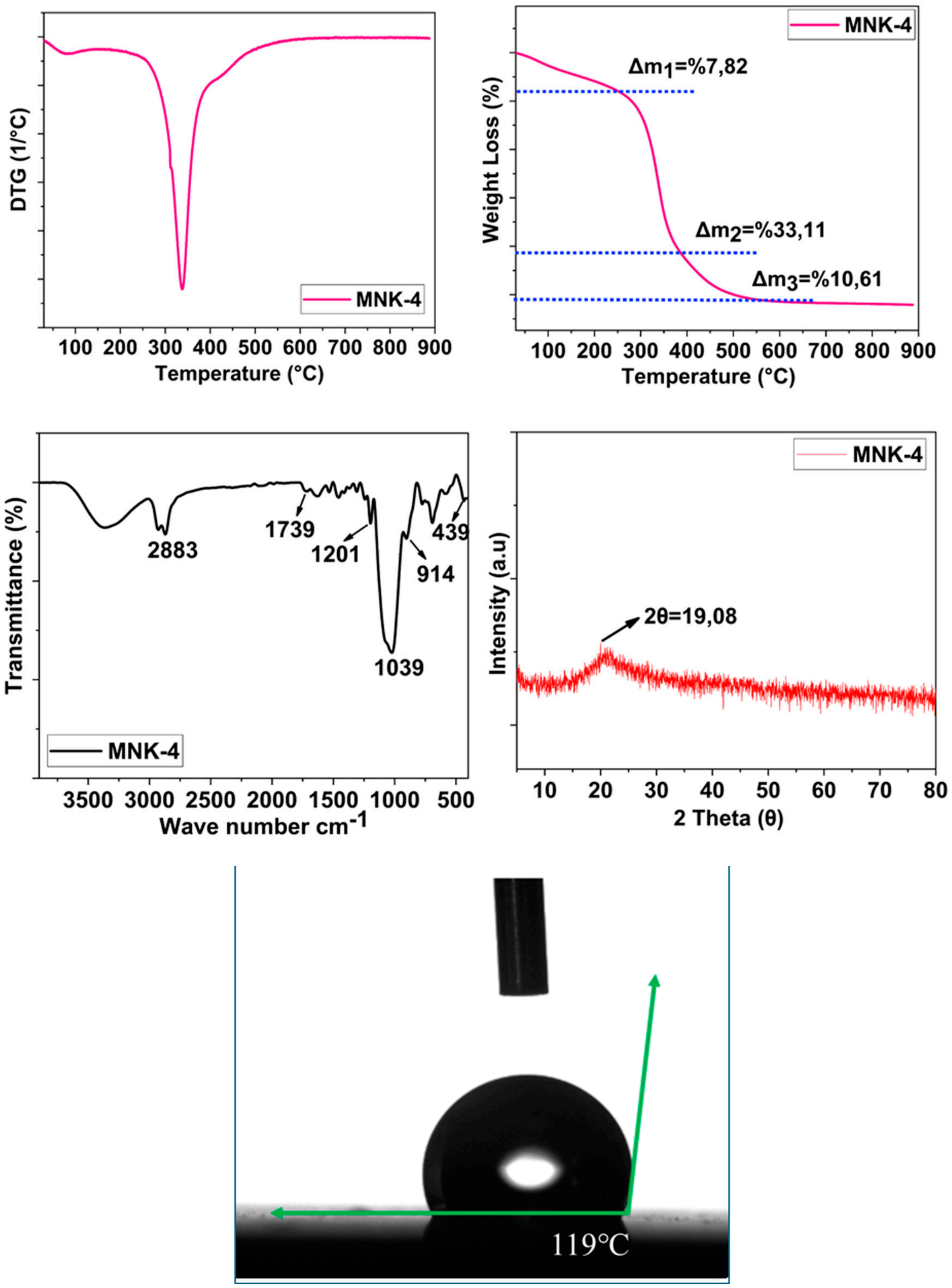

Figure S6: TG-DTA investigation, FT-IR spectrum and XRD diffraction results with water contact angle of the MNK-4 coating

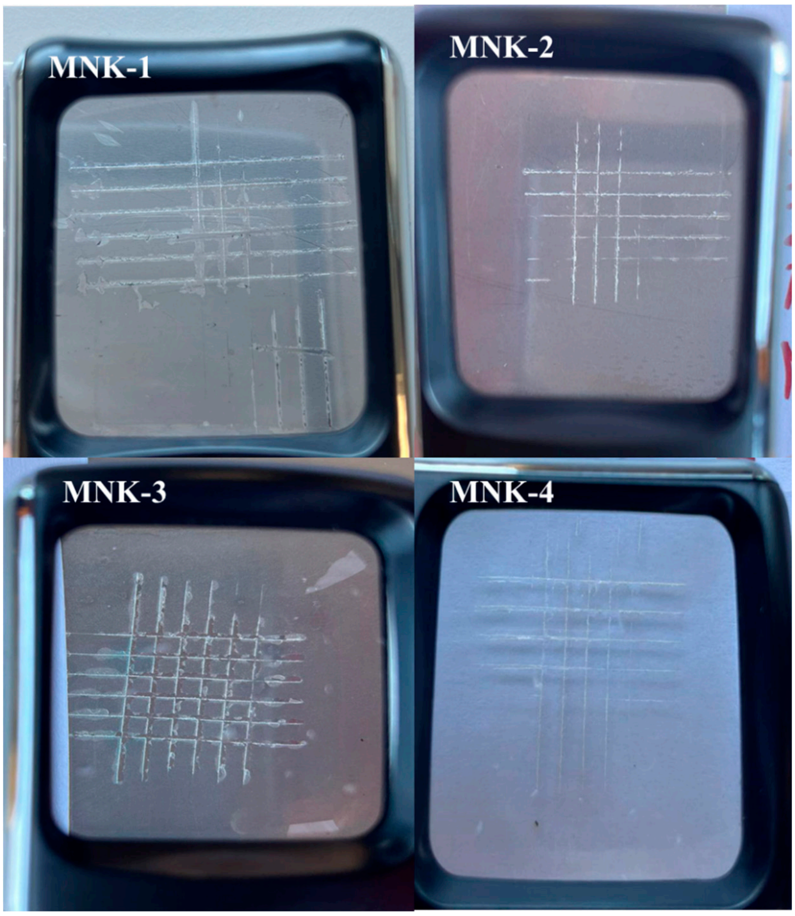

Figure S7: Cross cut test of the MNK-1, MNK-2, MNK-3 and MNK-4 coatings
